# The Predicted Potential Impact of COVID-19 Pandemic on Tuberculosis Epidemic in Tamil Nadu, South India

**DOI:** 10.3390/tropicalmed9010012

**Published:** 2024-01-04

**Authors:** Malaisamy Muniyandi, Karikalan Nagarajan, Kavi Mathiyazhagan, Prathiksha Giridharan, Kannan Thiruvengadam, Rajendran Krishnan

**Affiliations:** ICMR-National Institute for Research in Tuberculosis, Chennai 60031, India

**Keywords:** tuberculosis, COVID-19, prevalence, incidence, mathematical modelling, Tamil Nadu

## Abstract

Objective: To estimate the prevalence and incidence of TB before and during the COVID-19 pandemic in Tamil Nadu, south India. Methods: In the present study, the effect of COVID-19 epidemiology on the TB epidemic was assessed by the SEIR (Susceptible-Exposed-Infected-Recovered), a compartmental epidemiological model. The model input parameters on compartments of TB and incidence of COVID-19 were collected from the published literature. Based on the data collected, point prevalence and incidence of TB per 100,000 population is calculated with and without COVID-19. A prediction was conducted up to 2025, trend analysis was performed, and a trend chi-square test and chi-square test of independence were used to test the difference between the prevalence with and without COVID-19. R software 2000 (R 4.0.0) was used for analysis. Results: The TB prevalence without and with COVID-19 decreases from 289 in 2020 to 271 in 2025 and from 289 in 2020 to 269 in 2025, respectively. Similarly, the incidence of TB was decreasing from 144 in 2020 to 135 in 2025 without COVID-19 and 143 in 2020 to 134 in 2025 with COVID-19. Though the TB burden is decreasing over the years, the trend was not statistically significant (*p* > 0.05). With respect to the district level, the prevalence and incidence of TB with and without COVID-19 is also found to be decreasing over the years. It was also found that the difference in the prevalence and incidence of TB with and without COVID-19 was not statically significant. Conclusion: The results of our study shows that there was an annual decline of around 2% from 2020 to 2025 in the trend of the prevalence and incidence of TB with and without COVID-19. Overall, there is a reduction, but it was not significant, and there is no significant effect of COVID-19 on TB in Tamil Nadu.

## 1. Introduction

The new coronavirus (COVID-19) pandemic is a major source of disaster in the 21st century, and it has caused enormous health, demographic, economic, and social impacts [1]. The global spread of COVID-19 has led to an unprecedented response from public health officials, governments, and individuals around the world. It includes measures such as travel restrictions, quarantine, social distancing, and vaccination campaigns being implemented in an attempt to slow down and control the spread of the virus. Many aspects of public health, particularly efforts to prevent and control tuberculosis (TB), were impacted by the COVID-19 epidemic. This has caused a shift in economic and political priorities towards COVID-19 containment, leading to a potential decrease in efforts to tackle TB [1].

Any connection between COVID-19 and TB is especially important for the Indian public health system because India is one of the main contributors to the burden of tuberculosis, with the greatest number of cases globally [2]. India has a significant number of people with a latent TB infection (LTBI) and a large burden of active TB cases, making it a major concern for public health. Social and economic factors have a significant impact on tuberculosis epidemiology, making prevention, treatment, and control more difficult [3]. The significant temporary alterations that could impact the TB epidemiology are lockdown-related disruptions such as pre-care-seeking patient delay, transportation of the heath facilities, delay in treatment completion, reduced laboratory capacity and health care staff, drug stock outs, supply interruptions, and usage of TB diagnostic tools for COVID-19. Additionally, it prevented undernutrition in the impoverished, lockdown-induced under-detection of active TB, and poverty that was exacerbated by transmission [4].

The World Health Organization (WHO) has provided guidance on how to address the impact of COVID-19 on TB [5]. WHO suggests leveraging the expertise of National TB Elimination Programmes (NTEP) for rapid testing and contact tracing for COVID-19 response. The use of digital technologies for remote care and support for people with TB is also encouraged. In response to the pandemic, public health officials and governments have implemented a range of measures in an effort to prevent the virus’s spread and mitigate its impact, including travel restrictions, quarantine and isolation, social distancing, masks and personal protective equipment, testing and contact tracing, and vaccination campaigns. These changes might have helped in the reduction of TB transmission. 

Lockdowns and times of high COVID-19 incidence and hospital saturation have been linked to a decrease in the case notification ratio, which is the main and immediate effect of COVID-19 spreading onto TB transmission dynamics [6]. We estimate that even before the more complicated and unpredictable consequences of the COVID-19 pandemic on TB management and transmission dynamics can be properly defined, this disruption alone will cause an increase in the TB burden in the coming years. For instance, sharp declines in the laboratory capacity required to enable a tuberculosis diagnosis are anticipated, as well as disruptions in the drug supply, which may lead to drug shortages and postpone the commencement of treatments until the supply chain is restored [7,8]. Although these measures have had significant impacts on the TB reduction rate, simultaneously, they have also had impact on the TB increase rate during the COVID-19 pandemic. In line with this, the current study aimed to provide a comprehensive understanding of the epidemiological impact of COVID-19 on the TB epidemic in Tamil Nadu using the SEIR (Susceptible-Exposed-Infected-Recovered), a compartmental epidemiological model. 

## 2. Materials and Methods

### 2.1. Study Area

Our study focuses on all districts of Tamil Nadu to assess the impact of COVID-19 on TB in Tamil Nadu, which is the tenth-largest state by area and the sixth-most populated state in India.

### 2.2. Study Population

The current study focuses on TB among the adult population (people who are 18 years of age or older) in Tamil Nadu.

### 2.3. Study Design

**Figure 1:**
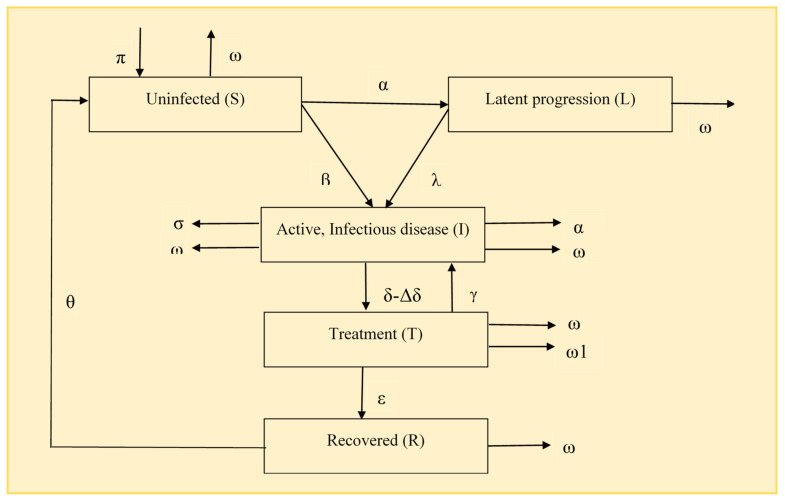
Model for the prevalence and incidence of TB with and without the COVID-19 pandemic in Tamil Nadu.

We used a mathematical modelling approach to estimate the prevalence and incidence of TB before and during the COVID-19 pandemic in all districts of Tamil Nadu. In this model, we have used the compartmental model SEIR, which is an extended version of the SIR model. The SIR model is a commonly used compartmental model that consists of three compartments, namely, (1) susceptible, (2) infected, and (3) recovered [9]. The SIR model uses a set of differential equations that consider the rates of infection, recovery, and death in order to analyze the dynamics of disease transmission. In the present study, we have further developed the SIR model by adding two more compartments, namely, (4) latent TB and (5) treatment for TB (Figure 1). For the SIR model with COVID-19, we have added two additional parameters, such as (1) lockdown (σ) and (2) mask utilization (σ1) to assess their impact on the TB burden. This has been added in the diagram below. From the active, infectious disease compartment, the two parameters lockdown and mask utilization have decreased with respect to their proportion.

The ordinary differential equations, which represent the extended SIR model (SEIR) with the impact of COVID-19, used in this study are:

(1) $$\frac{dS}{dt}=-\left(\alpha \times S\right)-\left(\beta \times S\right)+\left(\theta \times R\right)+\left(\pi \times N\right)-\left(\omega \times S\right)$$

(2) $$\frac{dL}{dt}=\left(\alpha \times S\right)-\left(\lambda \times L\right)-\left(\omega \times L\right)$$

(3) $$\frac{dI}{dt}=\left(\beta \times S\right)+\left(\lambda \times L\right)-\left(\left(\delta -∆\delta \right)\times I\right)+\left(\gamma \times T\right)-\left(\omega \times I\right)-\left(\omega_{1}\times I\right)-(\sigma \times I-\left(\sigma_{1}\times I\right))$$

(4) $$\frac{dT}{dt}=\left(\delta \times I\right)-\left(\omega \times T\right)-\left(\omega_{1}\times T\right)-\left(\gamma \times T\right)-(\varepsilon \times T)$$

(5) $$\frac{dR}{dt}=(\varepsilon \times T)-\left(\theta \times R\right)-\left(\omega \times R\right).$$

The two mathematical models are used to estimate the prevalence and incidence over the period 2017–2025 (without COVID-19) and 2020–2025 (with COVID-19). For the analysis, R software 2000 (R 4.0.0) is used. This model is modified by adding exposed and treatment compartments to account for a change in the overall population.

### 2.4. Input Parameter

Table 1 shows the input parameters used in the SEIR model. The parameters are uninfected to latent progression (*α*) [10], new susceptible (π) [11], uninfected to active infection (*β*) [12], latent progression to active infection (*λ*) [13], active infection to treatment (*δ*) [14], treatment to active infection (*γ*) [15], treatment to recovered (*ε*) [11], recovered to uninfected (*θ*), all-cause mortality ($\omega )\left[10\right],$ TB mortality (ω1) [11], lockdown (σ), and mask utilization (σ1). The values of these parameters were collected from the published literature. 

### 2.5. Data Collection

#### 2.5.1. TB Prevalence

The prevalence of TB has been collected over the period 2017 to 2021. The data was collected with a focus on various factors, including the study area (rural or urban), study setting (national survey, community survey, and household survey), sample size, and the method used to diagnose TB (culture or smear) [9,10,11,16]. The TB period prevalence of 301 per 100,000 population is considered as the baseline for the year 2017 [8].

#### 2.5.2. TB Incidence

Similarly, the incidences of TB in Tamil Nadu were collected from various published literature. The data were collected with a focus on the study area, study design (prospective cohort, community survey, household survey, and national survey), and sample size [17,18,19].

#### 2.5.3. COVID-19

Data on confirmed, recovered, and death due to COVID-19 in Tamil Nadu from March 2020 to November 2022 were collected from the Health and Family Welfare Department, Government of Tamil Nadu, Stop Corona TN site [20]. These data were analyzed to understand the impact of COVID-19 on the state of Tamil Nadu and their possible impact on TB.

#### 2.5.4. Mortality

Our study involved collecting data on the mortality due to TB in Tamil Nadu. The data were collected from the different sources, such as Statista and NTEP report of 2021. The data collection process involved analyzing the TB mortality rate in both rural and urban areas [21].

#### 2.5.5. Mask Utilization

Several studies have found that the use of masks can significantly reduce the transmission of TB [22,23]. It was also recommended that the patients with infectious TB wear a mask to prevent TB transmission in the community [24]. However, it should be noted that it cannot be assumed that all household contacts wear masks at all times. As a result, this study has assumed a marginal impact on transmission probability of 0.005. 

### 2.6. Data Analysis

In this study, SEIR was used to estimate the prevalence and incidence of TB before and during the COVID-19 pandemic. The model was developed from the basic framework of the SIR model. The extended SEIR model is derived to reflect the epidemiological conditions and respective care cascades in Tamil Nadu, both before and during the COVID-19 pandemic. 

### 2.7. SEIR Model for TB Prevalence

Based on actual data, we estimated the prevalence over the period from 2017 to 2025 before and during the COVID-19 pandemic. By comparing the prevalence rates between these two time periods with and without COVID-19, the difference in prevalence was quantified. Additionally, the prevalence has been predicted for all districts of Tamil Nadu in the same period. The following formula is used to calculate the prevalence:

(6) $$\mathrm{Prevalence}\;\mathrm{of}\;\mathrm{nth}\;\mathrm{year}=\frac{\mathrm{Infection}\;\mathrm{of}\;nth\;\mathrm{year}\;+\;\mathrm{Infection}\;\mathrm{of}\;(n-1)\mathrm{th}\;\mathrm{year}}{Total\;Population\;of\;nth\;year}*100,000.$$

We have derived six ordinary differential equations (ODEs) for the SEIR model that can be implemented to present the impact of the period before and during COVID-19 on TB. The prevalence rate was calculated per 100,000 population from the outputs of total population, susceptible cases, latent progression, active infectious cases, treatment, recovery, and mortality (Table 2) for the SEIR model. 

### 2.8. Impact of COVID-19 on Prevalence and Incidence

To find the impact of COVID-19 on TB prevalence and incidence in Tamil Nadu, we included a marginal presumption of mask utilization and social distance as an intervention of COVID-19 in this model during the years 2020 to 2025.

### 2.9. SEIR Model for TB Incidence 

Using the SEIR model, we estimated the incidence without COVID-19 for the period from 2017 to 2025 and with COVID-19 for the period from 2020 to 2025. The calculated incidence rate was derived from the model output. It was measured as the number of cases per 100,000 population. The below formula is used to calculate TB incidence by using the SEIR model.

(7) $$\mathrm{Incidence}\;\mathrm{of}\;\mathrm{nth}\;\mathrm{year}=\frac{Infection\;of\;nth\;year}{Total\;Population\;of\;nth\;year}*100,000$$

## 3. Results

### 3.1. SEIR Model Values for with and without the Impact of COVID-19

Using the SEIR model, the values for the total population greater than 18 years of age, total susceptible cases, patients with LTBI, patients with active TB infection, patients who undergo treatment, patients recovered after the treatment, and mortality due to TB are estimated for with and without the impact of COVID-19 (Table 2). The total population is increasing as the year increases. As SEIR is a compartmental model, patients are moving from one compartment to another. Approximately 71% of the patients from the total population are moving to the susceptible population. Around 40% of the patients from the susceptible population are moving to the LTBI compartment, 0.5% of the LTBI patients are moving to the active TB compartment, and 83% of the patients are moving from the active TB infection compartment to recovery compartment. Among the patients from the active TB compartment, 13% are moving to the TB mortality compartment. 

### 3.2. Prevalence of TB without the Impact of COVID-19

**Figure 2:**
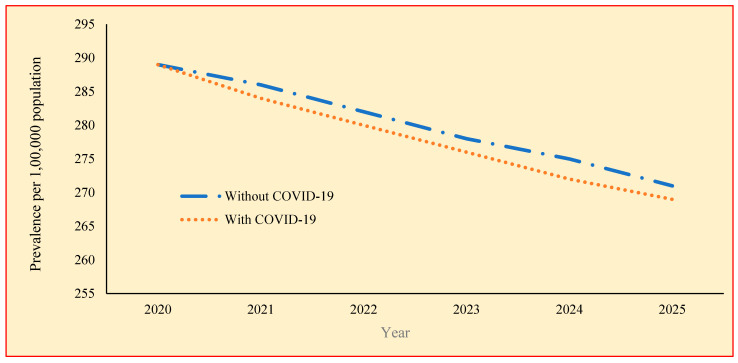
TB prevalence without and with the impact of COVID-19.

**Figure 3:**
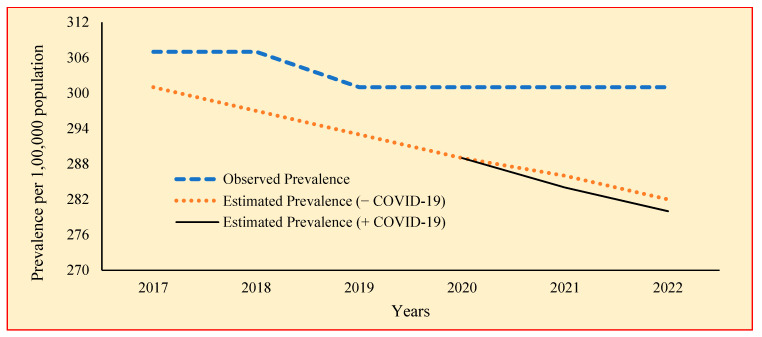
Observed prevalence rate and estimated prevalence rate on TB (without/with COVID-19) in Tamil Nadu from the year 2017 to 2022.

Table 3 shows the prevalence of TB with and without the impact of COVID-19 calculated for the years 2017 to 2025 using the model parameters. The highest prevalence without the impact of COVID-19 is found to be 289 in the year 2020, ranging from 271 to 289 per 100,000 population. The prevalence gradually decreases over the years from 2017 to 2025 (Figure 2). The rate of reduction is found to be similar in all the years. The test showed that there is no significant difference between the TB prevalence over the years without the impact of COVID-19. Figure 3 shows the observed and estimated prevalence of TB per 100,000 population for with and without COVID-19.

### 3.3. Prevalence of TB with the Impact of COVID-19

The highest prevalence with the impact of COVID-19 is found to be 289 in the year 2020, ranging from 269 in 2025 from 289 in 2020. With COVID-19, the prevalence gradually decreases from 289 to 269 over the years 2020–2025. Though the prevalence is lower with the impact of COVID-19, there is no statistically significant difference between with and without the impact of COVID-19 (*p* = 0.314).

### 3.4. Incidence of TB without the Impact of COVID-19

**Figure 4:**
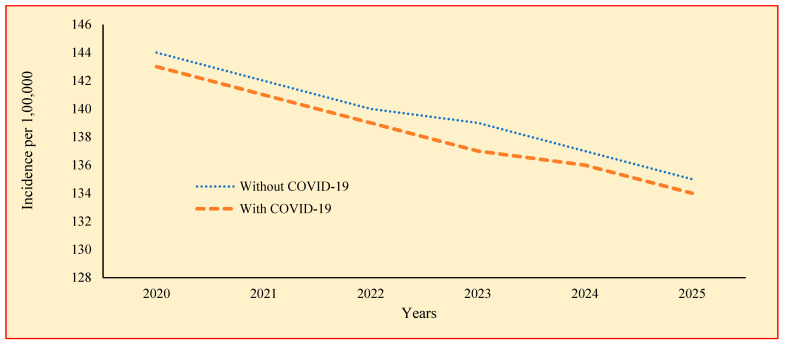
TB incidence without and with the impact of COVID-19 during the years 2020 to 2025.

Table 4 shows the incidence of TB with and without the impact of COVID-19. The highest incidence of TB with the impact of COVID-19 is found to be 143 in 2020, ranging from 134 in 2025 to 143 in 2025. Similar to prevalence, incidence is also showing a decline over the years. The incidence of TB with the impact of COVID-19 is comparatively less than the incidence of TB without the impact of COVID-19. This is visualized in a graph in Figure 4. The difference in the reduction rate gradually increases over the years from 0.598 to 0.877 per 100,000 population. 

### 3.5. Incidence of TB with the Impact of COVID-19

Table 4 gives the incidence of TB with the impact of COVID-19. The incidence is found to be highest in the year 2020 (143), ranging from 134 in 2025 to 143 in 2020. The prevalence of TB with COVID-19 is found to be comparatively lower than the prevalence of TB without COVID-19. Though there is a reduction, the test showed that there is no significant difference between the TB incidences over the years with the impact of COVID-19. 

### 3.6. District-Wise Prevalence of TB without the Impact of COVID-19

Table 5 shows the overall, the district-wise prevalence also decreases over the years. The lowest and highest prevalence is found to be 183 in Perambalur and 323 in Chennai in the year 2025 and 2020, respectively. The lowest prevalence is found to be in 183 in Perambalur in the year 2025. The second highest prevalence is 311 in Vellore. 

### 3.7. District-Wise Prevalence of TB with the Impact of COVID-19

The prevalence with the impact of COVID-19 is found to be very high in Chennai, ranging from 144 to 154. It is followed by Vellore, ranging from 139 to 148. The lowest prevalence without the impact of COVID-19 is found in the district Nilgiris, ranging from 84 to 93 per 100,000 population. When compared to the prevalence rates without the impact of COVID-19, the prevalence in districts with the impact of COVID-19 is low. 

### 3.8. District-Wise Incidence of TB without the Impact of COVID-19

Table 6 displays the district-wise incidence of TB with and without the impact of COVID-19. Without the impact of COVID-19, the incidence of TB is found to be very high in Chennai, ranging from 145 in 2020 to 161 in 2025. The lowest prevalence is found in the district Perambalur, ranging from 91 to 101. The second highest prevalent district is found to be Vellore, ranging from 140 to 155, followed by Kanchipuram. The lowest incidence without the impact of COVID-19 is found to be 91 in Perambalur in the year 2022.

### 3.9. District-Wise Incidence of TB with the Impact of COVID-19

The lowest incidence of TB with the impact of COVID-19 found in the district Nilgiris is 84 in the year 2025. The highest incidence is 154 in Chennai in 2020, ranging from 144 in 2025 to 154 in 2025. The incidence of TB with the impact of COVID-19 is found to be comparatively less than the incidence of TB without the impact of COVID-19, though the difference is not significance.

## 4. Discussion

The salient finding from this study was that, overall, the prevalence and incidence of TB is in a declining trend during 2017 to 2025 in Tamil Nadu. We have attempted to study the impact of COVID-19 on the TB burden in terms of prevalence and incidence. We found that there is no negative impact due to COVID-19 on the TB burden in Tamil Nadu. However, it was estimated that there is a positive impact on the declining trend of the TB burden during COVID-19 in Tamil Nadu. This declining trend might be a result due to the COVID-19 preventive measures, such as mask utilization, lockdowns, and social distancing. 

Our study substantiates the findings of other studies that the use of masks significantly reduced the transmission of TB among children and household contacts and MDR-TB among hospital wards. There are studies that reported a positive trend of TB has been reversed due to the COVID-19 pandemic outbreak. They expected that the unintended consequences of COVID-19 preventive measures such as restriction and reduced access to healthcare resulted in a drop in access to TB services [25]. The predictive models have also reported that the negative impact of COVID-19 on TB is much larger [26]. The other important issue is that during COVID-19, high deaths due to TB were reported globally [27]. The reason reported is the reduced access to TB care. However, our estimates showed that there is no negative impact on the TB burden in Tamil Nadu. During the COVID-19 pandemic, the government of Tamil Nadu diagnosed more TB cases through CT scans taken for COVID-19 diagnosis in the Makkalai Thedi Maruthuvam (MTM) program [28]. It is a special new flagship program, which has been implemented to provide doorstep healthcare facilities to eliminate the visit of poor people to various hospitals. NTEP supplied two months of anti-TB drugs to TB patients to continue the treatment during the lockdown period. These efforts would have contributed in maintaining the positive trend of the TB decline. 

In order to study the impact of the pandemic COVID-19 on TB in Tamil Nadu mathematically, this study has used a modified version of the SIR model termed the SEIR model, which has yielded the trend of the TB burden with and without the impact of COVID-19. The model was designed in such a fashion that it is able to yield the prevalence of TB with and without COVID-19 by tuning its parameters. The input parameters used in the model were taken from different data sources from published literature taken at different periods. The robustness of the model needs to be studied further, considering uncertainties of the parameter values with respect to time and place. 

A similar finding was observed in all the districts that there was no effect of COVID-19 on TB prevalence and incidence. It was found that among districts, Chennai has a higher prevalence and incidence compared to both with and without the COVID-19 pandemic. Since this is a metropolitan city with a high population density and mixture of various segments of the population, including slums, the high prevalence and incidence of TB in Chennai might be due to many migrants who have acquired TB infection, leading to an increased risk of developing an active TB disease. This is also due to the fact that more vulnerable groups for TB such as the poor and homeless are found living in Chennai [29,30,31,32]. They are at risk of TB infection and progression due to factors such as increased exposure and infection risks. 

In Tamil Nadu, TB case findings were low in the COVID-19 pandemic lockdown. The government of Tamil Nadu has taken efforts on targeted interventions to bring them back to normal after COVID-19. These were aimed at testing more for TB, along the same lines of COVID-19, to detect more TB cases and increased TB case findings [33]. In addition, the following measures are also to be taken to improve the case findings, such as enhanced active case finding through health care workers, mobile diagnostic vans, active surveillance, testing all TB suspected cases with any duration of cough, active screening of household contacts and vulnerable population, advocacy, and social mobilization [34].

Even though NTEP has evolved significantly since it was first implemented and has experienced significant changes in recent years, significant work is still being conducted to improve the program’s focus on patients and the ability to offer complete treatment, care, and support [35]. In order to end TB by 2025, a number of steps have been taken, including: (1) treating tuberculosis (TB) appropriately to stop drug resistance from developing and break the chain of transmission; (2) increasing capacity for ongoing surveillance; and (3) stopping the spread of LTBI and TB infection. Since it was not able to have met the objectives by 2020, the new National Strategic Plan (NSP) was launched in 2020. Even though there are now complex and efficient strategies and technology for the diagnosis, treatment, and care of TB, additional work needs to be done in order to accelerate the state of Tamil Nadu’s TB incidence decline [29].

There are several other studies that assessed the impact of the COVID-19 pandemic on TB. Anurag Bhargava et al. has concluded that COVID-19 induced shock, and this could significantly affect the incidence and mortality of tuberculosis and take a long time to return to normal [36]. Lucia Cilloni et al.’s study on the impact of the COVID-19 pandemic on the TB epidemic by a modelling analysis has revealed that three key countries illustrated that even short COVID-19-related lockdowns can generate long-lasting setbacks in TB control [34]. In our study, we attempted to study the TB prevalence amongst all populations from across the state of Tamil Nadu. The results of this study provide vital information on the TB disease situation amongst this population and can serve as baseline data for a future evaluation of the impact of disease control measures and epidemiological trends. From the SEIR model, we estimated that the TB prevalence and TB incidence from 2017 to 2025 in Tamil Nadu has been decreasing by around 2% annually. This may be primarily attributable to a number of strict actions taken during the COVID-19 epidemic, as well as to people’s greater awareness of protection and protective measures, all of which reduced the risk of contracting tuberculosis. In addition, the COVID-19 pandemic changed people’s lifestyles by promoting mask use, hand washing, and social distancing. This implementation of preventative measures against the transmission of COVID-19 may have had a significant impact on reducing respiratory infections, including TB. By applying similar approaches to other diseases, such as TB, it is possible to improve detection and reduce the burden of illness in the population. It is important for health systems to remain vigilant and adaptable in the face of emerging threats to ensure the best possible outcomes for patients and communities.

Finally, we note that there are certain drawbacks with our technique that also affect TB transmission models. As an example, our model’s result is contingent upon a number of epidemiological characteristics and starting burden estimates that are unknown, which introduce uncertainty into the findings. This implies that, as with any other model that depends on the input data, future advancements in measuring it should also have an effect on the quantitative results of our mathematical model.

## 5. Limitations

As this study was carried out among adults (≥18 years of age), our focus was only on the TB prevalence and incidence. The model only considers susceptibility, infectiousness, and recovery; the other factors such as age, immunity, and genetics are not considered. Additionally, the model is short term and not suitable for predicting long-term epidemic evolution. The additional disruptions that might have been caused due to COVID-19 on the TB epidemic, such as the delay in diagnosis, impact of COVID-19 vaccination, and reduced access to healthcare, has not been considered in this study. 

## 6. Conclusions

This study has reflected that there are not many changes in TB that resulted from the COVID-19 pandemic. The impact of the COVID-19 pandemic and the estimated TB prevalence and incidence in Tamil Nadu are in a reducing trend from 2020 to 2025. The reducing trend is not significant, despite the various efforts made by the government to accelerate the reduction. The findings of the study provide a reliable estimate of the trend of pulmonary TB among the adult population in Tamil Nadu during the COVID-19 pandemic. Currently, the NTEP have also been making efforts to introduce interventions that are more preventive for the TB disease and latent TB infection like BCG revaccination, TB preventive therapy for children and adults, active case finding, and nutrition supplementation to speed up the reduction of TB prevalence and incidence in Tamil Nadu. Though there are various measures being taken up for the acceleration of the reduction of TB cases, there is still a need to focus on accelerating the process. 

## Figures and Tables

**Table 1:** Description of model parameters.

Parameter	Model Parameter Name	Values	References
*α*	Uninfected to Latent Progression	0.33	8
π	New susceptible	0.0123	9
*β*	Uninfected to Active infection	0.00301	10
*λ*	Latent Progression to Active infection	0.005	9
*δ*	Active infection to Treatment	0.96	11
*γ*	Treatment to Active infection	0.102	12
*ε*	Treatment to Recovered	0.83	12
*θ*	Recovered to Uninfected	0.99	Assumption
ω	All-cause mortality	0.009	10
ω _1_	TB mortality	0.059	12
σ	Lockdown	0.005	Assumption
σ _1_	Mask Utilization	0.005	Assumption

**Table 2:** SEIR model values for with and without the impact of COVID-19.

**SEIR Model Values for without the Impact of COVID-19**							
**Year**	**Population > 15**	**Susceptible**	**LTBI**	**Active TB**	**Treatment**	**Recovery**	**Mortality**
2017	57,068,340	39,677,931	17,138,298	85,543	91,614	74,954	10,440
2018	57,342,263	40,097,166	16,993,397	84,842	91,262	75,596	10,390
2019	57,616,352	40,516,263	16,849,722	84,132	90,671	75,565	10,313
2020	57,890,625	40,934,751	16,707,262	83,423	89,980	75,209	10,231
2021	58,165,093	41,352,415	16,566,006	82,718	89,251	74,703	10,146
2022	58,439,764	41,769,162	16,425,945	82,019	88,510	74,129	10,061
2023	58,714,646	42,184,958	16,287,068	81326	87,767	73,528	9976
2024	58,989,745	42,599,798	16,149,365	80,638	87,027	72,917	9892
2025	59,265,069	43,013,688	16,012,827	79,956	86,292	72,305	9809
**SEIR Model Values for with the Impact of COVID-19**							
**Year**	**Population > 15**	**Susceptible**	**LTBI**	**Active TB**	**Treatment**	**Recovery**	**Mortality**
2020	57,889,810	40,934,731	16,707,232	82,923	89,769	75,156	10,242
2021	58,163,505	41,352,253	16,565,917	82,063	88,783	74,490	10,080
2022	58,437,425	41,768,684	16,425,787	81,318	87,884	73,752	9983
2023	58,711,568	42,184,018	16,286,840	80,614	87,063	73,034	9893
2024	58,985,937	42,598,300	16,149,067	79,927	86,289	72,354	9807
2025	59,260,535	43,011,578	16,012,459	79,249	85,542	71,707	9723

**Table 3:** TB prevalence (without/with COVID-19).

Year	(Without COVID-19)		(With COVID-19)		Difference Rate (%)
	Prevalence/100,000 Population	Reduction (%)	Prevalence/100,000 Population	Reduction (%)	
2020	289 (283–296)	1.327	289 (283–294)	1.629	0.298
2021	286 (279–292)	1.329	284 (278–289)	1.732	0.697
2022	282 (276–288)	1.328	280 (274–285)	1.458	0.826
2023	278 (272–285)	1.327	276 (270–281)	1.368	0.867
2024	275 (268–281)	1.325	272 (267–278)	1.338	0.880
2025	271 (265–277)	1.323	269 (263–274)	1.327	0.884

**Table 4:** TB incidence (without/with COVID-19).

Year	(Without COVID-19)		(With COVID-19)		Difference Rate (%)
	Incidence/100,000 Population	Reduction (%)	Incidence/100,000 Population	Reduction (%)	
2020	144 (141–147)	1.312	143 (141–146)	1.902	0.598
2021	142 (139–145)	1.312	141 (139–144)	1.503	0.790
2022	140 (137–143)	1.311	139 (137–142)	1.372	0.851
2023	139 (135–142)	1.310	137 (135–140)	1.329	0.870
2024	137 (134–140)	1.308	136 (133–138)	1.313	0.876
2025	135 (132–138)	1.306	134 (131–136)	1.307	0.877

**Table 5:** District-wise TB prevalence with and without the impact of COVID-19 from 2017 to 2025.

S. No	District	Without Impact									With Impact					
		2017	2018	2019	2020	2021	2022	2023	2024	2025	2020	2021	2022	2023	2024	2025
1	Ariyalur	207	204	202	199	197	194	192	189	187	197	195	192	190	188	185
2	Chennai	323	319	315	311	307	303	299	295	291	308	304	300	296	293	289
3	Coimbatore	303	299	296	292	288	284	281	277	273	289	286	282	278	275	271
4	Cuddalore	287	284	280	276	273	269	266	262	259	274	270	267	264	260	257
5	Dharmapuri	235	232	229	226	223	220	218	215	212	224	221	219	216	213	210
6	Dindigul	259	256	253	249	246	243	240	237	234	247	244	241	238	235	232
7	Erode	263	260	257	253	250	247	244	240	237	251	248	245	241	238	235
8	Kanchipuram	311	307	303	300	296	292	288	284	281	297	293	289	285	282	278
9	Kanniyakumari	247	244	241	238	235	232	229	226	223	236	233	230	227	224	221
10	Karur	211	208	206	203	200	198	195	193	190	201	199	196	194	191	189
11	Krishnagiri	247	244	241	238	235	232	229	226	223	236	233	230	227	224	221
12	Madurai	299	296	292	288	284	281	277	273	270	285	282	278	275	271	267
13	Namakkal	243	240	237	234	231	228	225	222	219	228	225	222	219	217	214
14	Nagapattinam	239	236	233	230	227	224	221	219	216	232	229	226	223	220	217
15	Permbalur	203	200	198	195	193	190	188	185	183	194	191	189	186	184	182
16	Pudukottai	239	236	233	230	227	224	221	219	216	228	225	222	219	217	214
17	Ramanathapuram	223	220	217	215	212	209	206	204	201	213	210	207	205	202	200
18	Salem	303	299	296	292	288	284	281	277	273	289	286	282	278	275	271
19	Sivaganga	223	220	217	215	212	209	206	204	201	213	210	207	205	202	200
20	Thanjavur	267	264	260	257	254	251	247	244	241	255	252	248	245	242	239
21	The Nilgiris	207	204	202	199	197	194	192	189	187	186	182	179	175	172	168
22	Theni	219	216	213	211	208	205	203	200	198	209	206	204	201	198	196
23	Tiruchy	291	288	284	280	277	273	270	266	263	197	193	189	185	182	178
24	Thiruvallur	307	303	300	296	292	288	284	281	277	209	206	204	201	198	196
25	Thiruvarur	219	216	213	211	208	205	203	200	198	209	206	204	201	198	196
26	Thoothukodi	243	240	237	234	231	228	225	222	219	232	229	226	223	220	217
27	Tirunelveli	299	296	292	288	284	281	277	273	270	285	282	278	275	271	267
28	Tiruppur	283	280	276	273	269	266	262	259	255	270	267	263	260	257	253
29	Thiruvanamalai	275	272	268	265	261	258	255	251	248	263	259	256	253	249	246
30	Vellore	311	307	303	300	296	292	288	284	281	297	293	289	285	282	278
31	Villupuram	303	299	296	292	288	284	281	277	273	289	286	282	278	275	271
32	Virudhunagar	251	248	245	242	239	236	232	230	227	240	236	233	230	228	225

**Table 6:** District-wise TB incidence with and without the impact of COVID-19 from 2017 to 2025.

S. No	District	Without Impact									With Impact					
		2017	2018	2019	2020	2021	2022	2023	2024	2025	2020	2021	2022	2023	2024	2025
1	Ariyalur	103	102	100	99	98	97	95	94	93	98	97	96	95	93	92
2	Chennai	161	159	157	155	153	151	149	147	145	154	152	150	148	146	144
3	Coimbatore	151	149	147	145	143	142	140	138	136	144	142	140	139	137	135
4	Cuddalore	143	141	139	138	136	134	132	131	129	136	135	133	131	130	128
5	Dharmapuri	117	116	114	113	111	110	108	107	106	112	110	109	107	106	105
6	Dindigul	129	127	126	124	123	121	119	118	116	123	122	120	118	117	115
7	Erode	131	129	128	126	124	123	121	120	118	125	123	122	120	119	117
8	Kanchipuram	155	153	151	149	147	145	143	142	140	148	146	144	142	140	139
9	Kanniyakumari	123	121	120	118	117	115	114	112	111	117	116	114	113	111	110
10	Krur	105	104	102	101	100	99	97	96	95	100	99	98	96	95	94
11	Krishnagiri	123	121	120	118	117	115	114	112	111	117	116	114	113	111	110
12	Madurai	149	147	145	143	142	140	138	136	134	142	140	138	137	135	133
13	Namakkal	121	120	118	116	115	114	112	111	109	114	112	111	109	108	106
14	Nagapattinam	119	118	116	115	113	112	110	109	107	115	114	113	111	110	108
15	Permbalur	101	100	98	97	96	95	94	92	91	96	95	94	93	92	90
16	Pudukottai	119	118	116	115	113	112	110	109	107	114	112	111	109	108	106
17	Ramanathapuram	111	110	108	107	105	104	103	101	100	106	105	103	102	101	99
18	Salem	151	149	147	145	143	142	140	138	136	144	142	140	139	137	135
19	Sivaganga	111	110	108	107	105	104	103	101	100	106	105	103	102	101	99
20	Thanjavur	133	131	130	128	126	125	123	122	120	127	125	124	122	121	119
21	The Nilgiris	103	102	100	99	98	97	95	94	93	93	91	89	87	85	84
22	Theni	109	108	106	105	104	102	101	100	98	104	103	101	100	99	98
23	Tiruchy	145	143	141	140	138	136	134	132	131	98	96	94	92	90	89
24	Thiruvallur	153	151	149	147	145	143	142	140	138	104	103	101	100	99	98
25	Thiruvarur	109	108	106	105	104	102	101	100	98	104	103	101	100	99	98
26	Thoothukodi	121	120	118	116	115	114	112	111	109	115	114	113	111	110	108
27	Tirunelveli	149	147	145	143	142	140	138	136	134	142	140	138	137	135	133
28	Tiruppur	141	139	137	136	134	132	131	129	127	135	133	131	129	128	126
29	Thiruvanamalai	137	135	134	132	130	128	127	125	124	131	129	127	126	124	123
30	Vellore	155	153	151	149	147	145	143	142	140	148	146	144	142	140	139
31	Villupuram	151	149	147	145	143	142	140	138	136	144	142	140	139	137	135
32	Virudhunagar	125	123	122	120	119	117	116	114	113	119	118	116	115	113	112

## Data Availability

All data generated during this study are included in this published article.

## References

[1] Cheval S., Mihai Adamescu C., Georgiadis T., Herrnegger M., Piticar A., Legates D.R. (2020). Observed and Potential Impacts of the COVID-19 Pandemic on the Environment. Int. J. Environ. Res. Public Health.

[2] World Health Organization Global Tuberculosis Report—2019. Geneva. https://iris.who.int/bitstream/handle/10665/329368/9789241565714-eng.pdf?sequence=19.

[3] Raviglione M., Sulis G. (2016). Tuberculosis 2015: Burden, challenges and strategy for control and elimination. Infect. Dis. Rep..

[4] Lönnroth K., Williams B.G., Cegielski P., Dye C. (2010). A consistent log-linear relationship between tuberculosis incidence and body mass index. Int. J. Epidemiol..

[5] Lee Y., Raviglione M.C., Flahault A. (2020). Use of Digital Technology to Enhance Tuberculosis Control: Scoping Review. J. Med. Internet Res..

[6] World Health Organization (2021). Global Tuberculosis Report 2021.

[7] World Health Organization Regional Office for Europe (2020). Rapid Communication on the Role of the GeneXpertt® Platform for Rapid Molecular Testing for SARS-COV-2 in the WHO European Region (2020).

[8] Adepoju P. (2020). Tuberculosis and HIV responses threatened by COVID-19. Lancet HIV.

[9] Harko T., Lobo F.S., Mak M.K. (2014). Exact analytical solutions of the Susceptible-Infected-Recovered (SIR) epidemic model and of the SIR model with equal death and birth rates. Appl. Math. Comput..

[10] Saha S., Kumar A., Saurabh K., Shankar S.H., Kashyap A., Nischal N., Biswas A., Wig N. (2019). Current status of treatment of latent tuberculosis infection in India. Indian J. Med. Sci..

[11] Knomea Tamil Nadu Birth-Rate. https://knoema.com/atlas/India/ranks/Birth-rate.

[12] ICMR-National Institute for Research in Tuberculosis (NIRT), Chennai (2021). National TB Prevalence Survey (2019–2022). https://tbcindia.gov.in/WriteReadData/l892s/25032022161020NATBPSReport.pdf.

[13] Dhanaraj B., Papanna M.K., Adinarayanan S., Vedachalam C., Sundaram V., Shanmugam S., Sekar G., Menon P.A., Wares F., Swaminathan S. (2015). Prevalence and risk factors for adult pulmonary tuberculosis in a metropolitan city of South India. PLoS ONE.

[14] Dolla C.K., Dhanaraj B., Chandrasekaran P., Selvaraj S., Menon P.A., Thiruvengadam K., Krishnan R., Mondal R., Malaisamy M., Marinalk S.B. (2021). Prevalence of bacteriologically confirmed pulmonary tuberculosis and associated risk factors: A community survey in Thirvallur District, south India. PLoS ONE.

[15] Gopi P.G., Subramani R., Radhakrishna S., Kolappan C., Sadacharam K., Devi T.S., Frieden T.R., Narayanan P.R. (2003). A baseline survey of the prevalence of tuberculosis in a community in south India at the commencement of a DOTS programme. Int. J. Tuberc. Lung Dis..

[16] Kolappan C., Subramani R., Radhakrishna S., Santha T., Wares F., Baskaran D., Selvakumar N., Narayanan P.R. (2013). Trends in the prevalence of pulmonary tuberculosis over a period of seven and half years in a rural community in south India with DOTS. Indian J. Tuberc..

[17] Mazumdar S., Satyanarayana S., Pai M. (2019). Self-reported tuberculosis in India: Evidence from NFHS-4. BMJ Global Health.

[18] Jeyashree K., Thangaraj J., Rade K., Modi B., Selvaraju S., Velusamy S., Akhil S., Vijayageetha M., Rani D.S., Sabarinathan R. (2022). Estimation of tuberculosis incidence at subnational level using three methods to monitor progress towards ending TB in India 2015–2020. BMJ Open.

[19] Narayanan S., Das S., Garg R., Hari L., Rao V.B., Frieden T.R., Narayanan P.R. (2002). Molecular epidemiology of tuberculosis in a rural area of high prevalence in South India: Implications for disease control and prevention. J. Clin. Microbiol..

[20] National Health Mission-TN Stop Corona TN (Media Bulletin). National Health Mission-Tamil Nadu, Health and Family Welfare Department, Government of Tamil Nadu. https://stopcorona.tn.gov.in/.

[21] Statista Rural and Urban Death Rates of India by States. https://www.statista.com/statistics/616282/rural-death-rate-by-state-and-union-territory-india.

[22] Singh M., Mynak M., Kumar L., Mathew J., Jindal S. (2005). Prevalence and risk factors for transmission of infection among children in household contact with adults having pulmonary tuberculosis. Arch. Dis. Child.

[23] Dharmadhikari A.S., Mphahlele M., Stoltz A., Venter K., Mathebula R., Masotla T., Lubbe W., Pagano M., First M., Jensen P.A. (2012). Surgical face masks worn by patients with multidrug-resistant tuberculosis: Impact on infectivity of air on a hospital ward. Am. J. Respir. Crit. Care Med..

[24] Aggarwal A.N., Agarwal R., Dhooria S., Prasad K.T., Sehgal I.S., Muthu V. (2022). Impact of COVID-19 pandemic on tuberculosis notifications in India. Lung India.

[25] Zamparelli S.S., Mormile M., Zamparelli A.S., Guarino A., Parrella R., Bocchino M. (2022). Clinical impact of COVID-19 on tuberculosis. Infez. Med..

[26] Tovar M., Aleta A., Sanz J., Moreno Y. (2022). Modeling the impact of COVID-19 on future tuberculosis burden. Commun. Med..

[27] World Health Organization Tuberculosis Deaths and Disease Increase during the COVID-19 Pandemic. World Health Organization, Geneva 2022. https://www.who.int/news/item/27-10-2022-tuberculosis-deaths-and-disease-increase-during-the-covid-19-pandemic.

[28] Subramanian M. (2023). Policy Note 2023–2024, Demand No. 19: Makkalai Thedi Maruthuvam.

[29] Dolla C., Padmapriyadarsini C., Pradeep Menon A., Muniyandi M., Adinarayanan S., Sekar G., Kavitha D., Tripathy S.P., Swaminathan S. (2017). Tuberculosis among the homeless in Chennai city, South India. Trans. R. Soc. Trop. Med. Hyg..

[30] Thomas B.E., Adinarayanan S., Manogaran C., Swaminathan S. (2015). Pulmonary tuberculosis among tribals in India: A systematic review & meta-analysis. Indian J. Med. Res..

[31] Thomas B.E., Thiruvengadam K., Vedhachalam C., Rao V.G., Vijayachari P., Rajiv Y., V R., Bansal A.K., Indira Krishna A.K., Joseph A. (2021). Prevalence of pulmonary tuberculosis among the tribal populations in India. PLoS ONE.

[32] Indira Krishnan A.K., Joseph A. (2020). Prevalence of tuberculosis among tribes aged 15 and above in Tamil Nadu. A community based cross-sectional study. Indian J. Tuberc..

[33] Central TB Division (2017). National Strategic Plan for Tuberculosis Elimination 2017–2025.

[34] Bhargava A., Shewade H.D. (2020). The potential impact of the COVID-19 response related lockdown on TB incidence and mortality in India. Indian J. Tuberc..

[35] Khanna A., Saha R., Ahmad N. (2023). National TB elimination programme—What has changed. Indian J. Med. Microbiol..

[36] Cilloni L., Fu H., Vesga J.F., Dowdy D., Pretorius C., Ahmedov S., Nair S.A., Mosneaga A., Masini E., Sahu S. (2020). The potential impact of the COVID-19 pandemic on the tuberculosis epidemic a modelling analysis. EClinicalMedicine.

